# Enhancing cervical cancer diagnosis with ensemble learning and shark optimization algorithm: comparative study of CT and MRI in cervical cancer diagnosis

**DOI:** 10.3389/fonc.2025.1608386

**Published:** 2025-10-10

**Authors:** Eman Hussein Alshdaifat, Amer Mahmoud Sindiani, Salem Alhatamleh, Hamad Yahia Abu Mhanna, Rola Madain, Mohammad Amin, Majd Malkawi, Ameera Jaradat, Hanan Fawaz Akhdar, Hasan Gharaibeh, Fatimah Maashey, Latifah Alghulayqah

**Affiliations:** ^1^ Department of Obstetrics and Gynecology, Faculty of Medicine, Yarmouk University, Irbid, Jordan; ^2^ Department of Obstetrics and Gynecology, Faculty of Medicine, Jordan University of Science and Technology, Irbid, Jordan; ^3^ Computer Science Department, Faculty of Information Technology and Computer Sciences, Yarmouk University, Irbid, Jordan; ^4^ Department of Medical Imaging, Faculty of Allied Medical Sciences, Isra University, Amman, Jordan; ^5^ Department of Obstetrics and Gynecology, Faculty of Medicine, Jordan University of Science and Technology, Irbid, Jordan; ^6^ Department of Medicine, Faculty of Medicine, Jordan University of Science and Technology, Irbid, Jordan; ^7^ Physics Department, Imam Mohammad Ibn Saud Islamic University (IMSIU), Riyadh, Saudi Arabia; ^8^ Artificial Intelligence and Data Innovation Office, King Hussein Cancer Center, Amman, Jordan

**Keywords:** gynecologic oncology, cervical cancer, classification medical image, deep learning, computer aided diagnosis, MRI image, CT image

## Abstract

Cervical cancer, one of the most common female cancers, can be detected with computed tomography (CT) and magnetic resonance imaging (MRI). Computer-aided diagnosis (CAD) methods based on artificial intelligence have been widely explored to improve traditional screening methods for cervical cancer detection. This study aims to compare the accuracy of CT and MRI in diagnosing cervical cancer using a novel methodology that combines the Large Vision Model (LVM) and InternImage, which reduces the misclassification of cervical tumors, especially in benign and malignant cases. InternImage (based on InceptionV3) extracts pre-trained deep features, making it more sensitive to tumor-specific patterns. At the same time, LVM focuses on fine-grained spatial features, helping to classify early changes in cervical pathology. In the Shark Optimization Algorithm (SOA), the procedure dynamically selects the optimal weight parameter, avoiding overreliance on a single model. This application improves generalization across different CT and MRI datasets. The performance of the proposed model is evaluated on two new datasets, KAUH-CCTD and KAUH-CCMD, collected from King Abdullah University Hospital (KAUH) in Jordan. The proposed model classified images into three categories: benign, malignant, and normal. The proposed model achieved the best performance in diagnosing CT images, with an accuracy of 98.49%, while achieving an accuracy of 92.92% in diagnosing MRI images. CT imaging, especially MRI, can detect tumor extension into the cervical stroma, which could change treatment approaches. Additionally, imaging plays a crucial role in monitoring treatment and patient progress to detect early disease relapses.

## 1 Introduction

Cervical cancer is one of the most common tumors amongst women worldwide, ranked fourth according to the 2022 Global Cancer Observatory (1). With approximately 660,000 new cases and 350,000 deaths reported, it is the primary cause of cancer-related deaths in 25 countries, in terms of incidence and mortality. Due to a lack of funding and resources for public health infrastructure, prevalence and mortality rates are up to ten times higher in developing countries (2). These challenges impede access to preventive measures, early detection, and treatment compared to developed countries. However, with advances in diagnostic methods, there has been a leap in detecting cervical cancer early on, thus increasing survival rates (3).

Magnetic resonance imaging (MRI), incorporating T2-weighted imaging (T2WI), contrast-enhanced T1-weighted imaging (CE-T1WI), and diffusion-weighted imaging (DWI) (4), is the optimal imaging tool in staging cervical cancer according to the 2018 FIGO (International Federation of Gynecology and Obstetrics) guidelines (5). In addition, emerging evidence highlights the amplified role of computed tomography (CT) in this domain (6). A systematic review indicated that the specificity of both MRI and CT for detecting parametrial tissue invasion and lymph node involvement is comparable, at approximately 80% (7). Nevertheless, their sensitivity varies significantly. Pelvic MRI exhibits a higher sensitivity, with a 74% rate for detecting parametrial tissue invasion and 60% for identifying regional lymph node involvement. In contrast, pelvic CT exhibits lower sensitivity, measuring 55% and 43% for those respective parameters (8).

Considering these points, artificial intelligence (AI) has significant potential in oncology. AI-based algorithms resorting to deep learning techniques have shown substantial promise in analyzing screening and diagnostic methods. These systems can detect subtle patterns and abnormalities that are overlooked by human examiners by processing extensive datasets, thereby reducing diagnostic errors (9). The previous few years have seen extensive use of AI for tumor detection, including skin tumors (10) and imaging-based tumor diagnosis (11). Screening and early diagnosis of cervical cancer are the most important applications of AI, wherein it can contribute to the alleviation of medical resource shortages as well as enhanced diagnosis accuracy (12). This paper reflects upon recent advancements in AI technology and its utility in early cervical cancer diagnosis. It further debates existing barriers to AI application within the health field and suggests research opportunities for the future.

The main objective of this study is to develop a reliable model for cervical cancer diagnosis using MRI and CT data, leveraging two new datasets collected from King Abdullah University Hospital, as well as advances in deep learning and computer vision techniques. The main contributions of this research paper are:

- This study offers two new data sets from cervical CT and MRI scans gathered at King Abdullah University Hospital in Jordan to detect and diagnose cervical cancer.
- A hybrid technique combining InternImage and Large Vision Model (LVM) is presented in this paper. The findings of the two models were combined, and cervical MRI and CT scans were classified using the Shark Optimisation Algorithm (SOA) to increase classification accuracy.
- A comparison was made between MRI and CT scanning in cervical cancer diagnosis.
- A comprehensive analysis and evaluation of the proposed model was conducted on datasets and compared with a set of pre-trained models, and its performance was analyzed.

The following sections of this work are detailed as follows: Section 2 discusses the approaches used, a comprehensive description of the dataset, the hybrid approach combining LVM and InternImage, and the training protocols. Section 3 summarizes the results analysis, evaluating the performance of the proposed model in cervical cancer diagnostic tests. Section 4 discusses the most important studies in cervical cancer diagnosis, and Section 5 concludes with key findings and suggestions for future research.

## 2 Materials and methods

**Figure 1:**
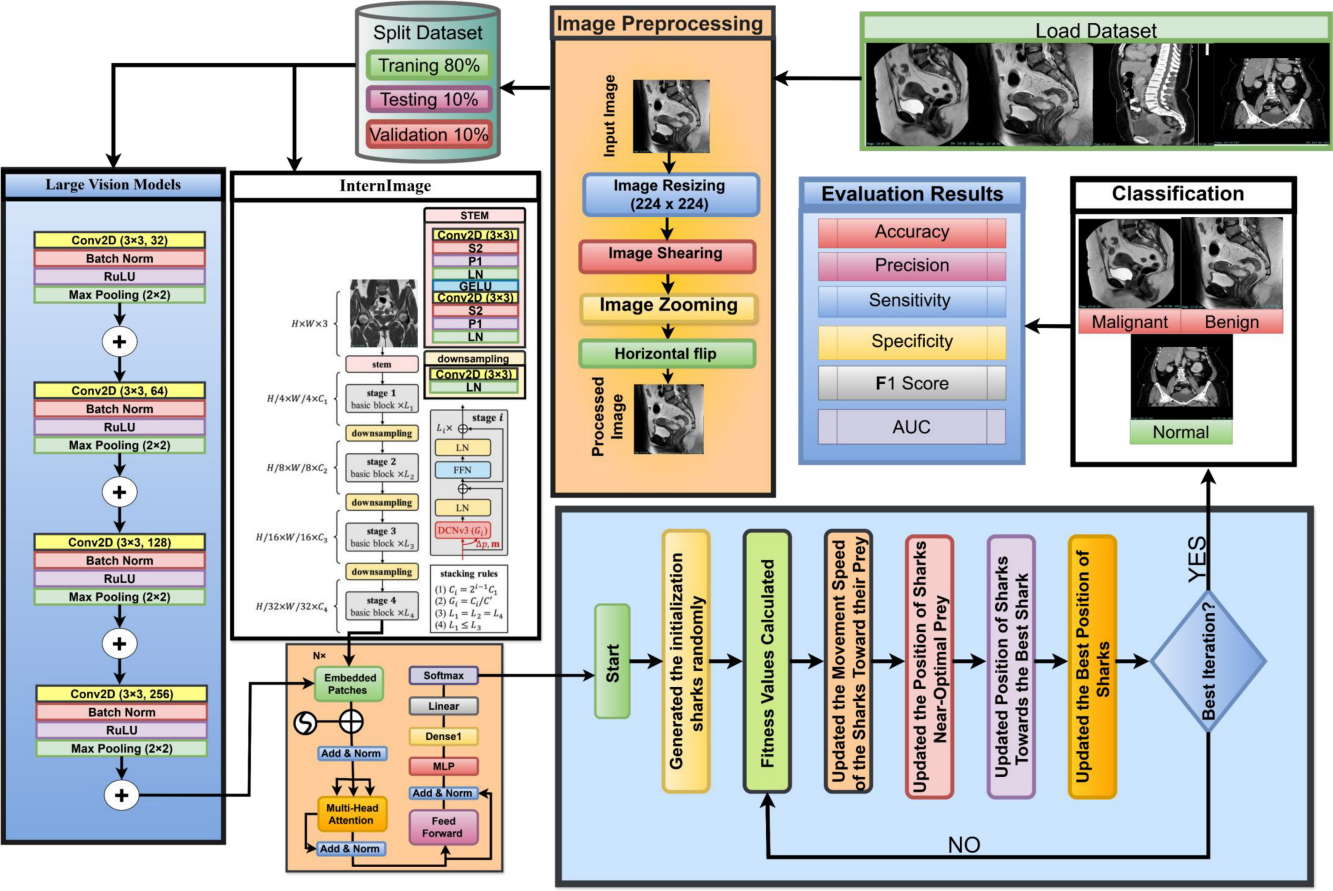
Process for methodology.

The proposed ensemble model is designed for cervical cancer diagnosis based on MRI and CT scans. As shown in **Figure 1**. The proposed model integrates the Large Vision Model (LVM) and the InternImage model, reducing misclassification of cervical tumors, especially benign and malignant cases. InternImage (based on InceptionV3) extracts pre-trained deep features, making it more sensitive to tumor-specific patterns. At the same time, the LVM model focuses on fine-grained spatial features, helping to classify early changes in cervical pathology. The Shark Optimization Algorithm (SOA) dynamically selects the optimal weight parameter, avoiding overreliance on a single model. This implementation enhances generalization across diverse MRI and CT datasets. Medical data, including MRI and CT images, is expected to vary in complexity, including resolution, contrast, and noise. These variations are learned from the ensemble, emphasizing high reliability during predictions in real-world clinical cases. Clinical impact, early detection, and accurate differentiation between normal, benign, and malignant conditions contribute to early diagnosis and treatment planning. Therefore, the accuracy of our comprehensive model reduces the likelihood of false negatives and ensures the identification of malignant conditions.

### 2.1 Dataset description (KAUH)

The records of 500 women who underwent cervical MRI and CT scans between early 2018 to late December 2024 were retrospectively reviewed. This study was conducted under guidelines and with institutional review board approval (IRB No. 21/171/2024) at King Abdullah University Hospital (KAUH), Jordan University of Science and Technology (JUST), Jordan. Institutional review board approval was granted. The data collection period spanned five months, from October 2024 to February 2025. The KAUH-CCMD and KAUH-CCTD datasets consist of three sections (sagittal, coronal, and axial) classified by hospital physicians as normal, benign, or malignant.

**Figure 2:**
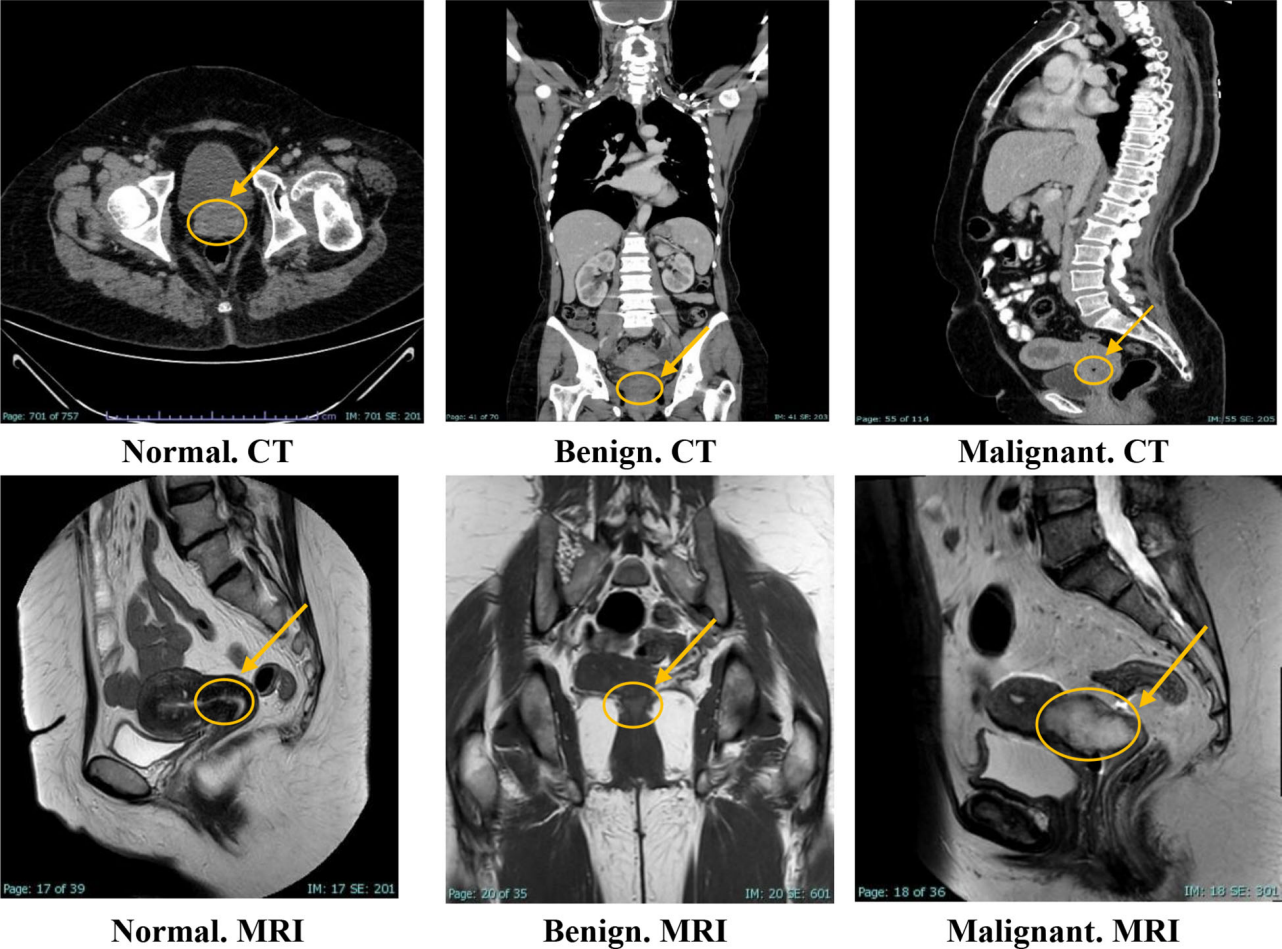
Sample from the KAUH-CCTD and KAUH-CCMD datasets.

The KAUH-CCMD dataset includes MRI scans captured using an Ingenia Ambition 1.5T scanner. The KAUH-CCTD dataset includes CT scans stored on a Philips Brilliance 64-channel CT scanner. Both datasets contain 1,974 images each, classified into three groups: normal, benign, and malignant. **Table 1** shows the distribution of the KAUH-CCTD and KAUH-CCMD datasets, while **Figure 2** provides a sample from each dataset.

**Table 1:** Distribution of images in each KAUH-CCTD and KAUH-CCMD category.

Dataset	Case	Images (n)
KAUH-CCTD	Normal	639
	Benign	680
	Malignant	655
	**Total**	**1974**
KAUH-CCMD	Normal	639
	Benign	680
	Malignant	655
	**Total**	**1974**

Bold values indicate the total number of images in each dataset.

### 2.2 Input image representation and preprocessing

Preprocessing medical images in machine learning and deep learning models is an important step, impacting the accuracy and generalization of the model (13). This study partitions the dataset into three subsets: 80% for training, 10% for testing, and 10% for validation. The detection of cervical cancer consists essentially of the analysis of medical images like, for instance, Pap smears or colposcopy images, to identify signs of possibly cancerous cells. The idea is to take meaningful features from these images to classify benign, malignant, or normal conditions. As far as image representation for cervical cancer detection is concerned, medical images have come to be represented as matrices (14), more specifically, as 3D tensors as shown in Equation 1:


(1) $$S\;\in \;{\mathbb{R}}^{H\times W\times C}$$

Where S input data, H is height (number of rows of pixels), W is width (number of columns of pixels), C is the number of color channels, which could vary with the imaging methodology (for example, RGB channels in colored images or gray levels in CT and MRI) (15).

Normalizing an input image would be vital for the stability and performance of deep learning models in training (16). For the case of cervical cancer detection incorporated into input images, associated with the pixel values from 0 to 255, it prompts that normalization be applied to scale the input into a more suitable range for the convergence of the model: Standardization could then be applied, where pixel values become normalized concerning the mean ( μ ) and standard deviation ( σ ) of the dataset as shown in Equation 2:


(2) $$S^{\prime }=\frac{S-\mu }{\sigma }$$

Medical imaging is characterized by limited datasets, thus making data augmentation necessary (17). Augmentation creates variations in training data, which equips the model with the ability to handle different variations (18). The model gets used to ingesting variations due to very slight rotations, size differences, and horizontal shifts. By rotating the image, the model learns to view cancerous cells from different angles. The rotation of angles is carried out by applying the rotation matrix $R_{\theta }$ , as shown in Equation 3. Scaling modifies image sizes, which is essential because cancerous cells may differ in size α is a randomly chosen scaling factor and thus allows the model to learn scale-invariant features using Equation 4. To avoid biasing the model towards one side, random horizontal flipping mirrors the image across its central axis, as described in Equation 5, while translation helps to adapt to object positioning. Shearing simulates distortions that render the model much stronger against variations in image quality.


(3) $$S_{new}^{'}=R_{\theta }\cdot S^{\prime }$$

(4) $$S_{new}^{'}=\alpha \cdot S^{\prime }$$

(5) $$S_{i,j}^{'}=S_{i,W-j}^{'}$$

### 2.3 Large vision model

**Figure 3:**
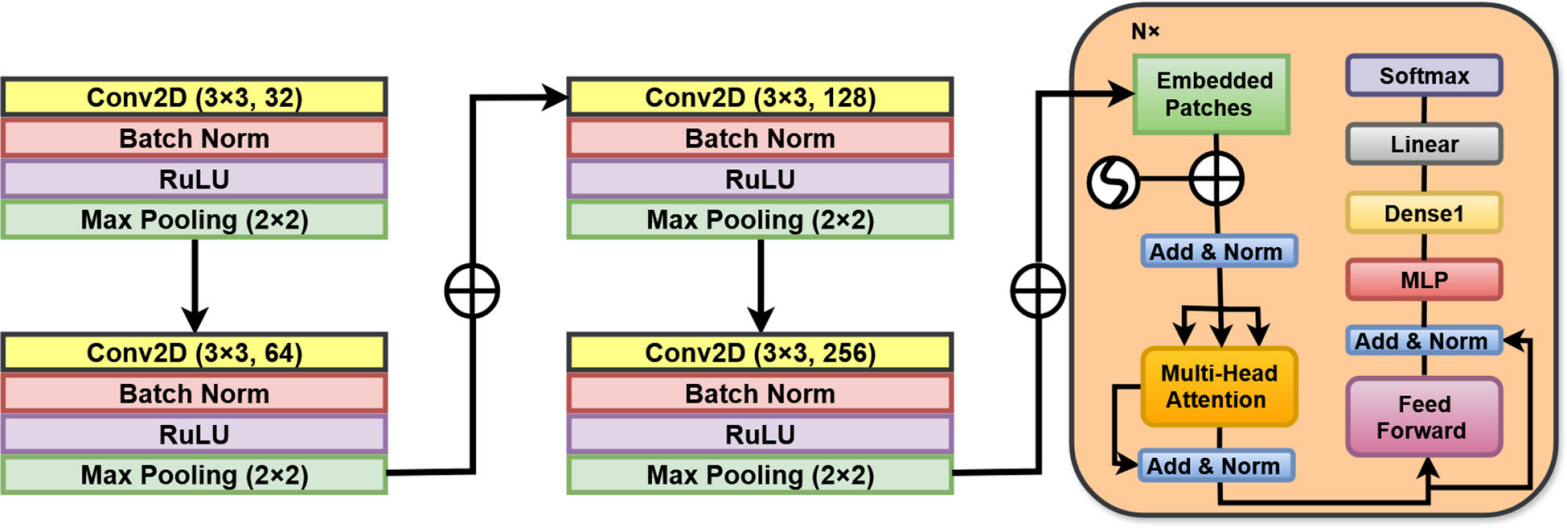
LVM methodology architecture.

Large Vision Models (LVMs) are a special kind of deep learning architecture designed to handle high-dimensional visual data. These models have achieved remarkable success in solving quite complicated image classification problems, especially concerning the medical imaging domain, with an even higher significance towards the diagnosis (19). LVMs apply some methods of building complex architecture using convolutional layers, attention mechanisms, and multi-scale feature extraction techniques for understanding complicated visual patterns such as those in cervical cancer detection scans. The inherent strength of LVMs is built on describing spatial hierarchies and representing long-range spatial dependencies in images. The conventional form of a convolutional neural network (CNN) focuses more on capturing sliding filters for local feature extraction, whereas transformer-type LVMs use self-attention for global contextual relationships throughout the image. Thus, fine-grained features, which could be essential in distinguishing normal, benign, and malignant cervical MRI and CT images, are indeed captured by the LVMs (19). **Figure 3** Illustrates the working architecture of the model.

In this sense, the LVMs hierarchically extract the image features (20), beginning from low features to the highest-level representation, low-level features are detected with the use of convolutional layers, followed by high-level features. The convolution operation in LVMs can be expressed as shown in Equation 6:


(6) $$F^{\left(l\right)}\left(i.j\right)=\left(\sigma \sum_{m}\sum_{n}W^{\left(l\right)}\left(m,n\right)\cdot F^{\left(l-1\right)}\left(i-m,j-n\right)+b^{\left(l\right)}\right)$$

Where $F^{\left(l\right)}\left(i.j\right)$ is the feature map at layer l at spatial location (i.j) , $W^{\left(l\right)}\left(m,n\right)$ is the filter weight of size (m×n) , $F^{\left(l-1\right)}\left(i-m,j-n\right)$ is the input feature map from the previous layer, $b^{\left(l\right)}\;$ is the bias term used, σ, is an activation function, such as rectified linear unit (ReLU). Usually, pooling layers follow the convolution operation in a deep learning neural network. Pooling reduces the spatial dimensionality of the feature maps while keeping relevant information. The max-pooling operation process as shown in Equation 7:


(7) $$P^{\left(l\right)}\left(i.j\right)=\begin{array}{c} max\\ \left(m,n\right)\epsilon R \end{array}\;\;F^{\left(l\right)}\left(i+m,j+n\right)$$

It consists of pooling windows R , $P^{\left(l\right)}\left(i.j\right)\;$ being a pooled feature at position (i.j) . This process improves computational efficiency and mainly focuses on the most meaningful activations to avoid overfitting (21). After the feature has been extracted from these high-level features, they are sent to fully connected layers where the classification decision is made.


(8) $$y_{k}=\frac{e^{z_{k}}}{\sum_{j}e^{z_{j}}}$$

Where $z_{k}$ denotes the logit score for class k , $y_{k}$ represents the probability of the input belonging to class k as shown in Equation 8. Now, the denominator ensures that the outputs get normalized to 1. Transformer-based LVMs such as Vision Transformers (ViTs) process images by cutting the images into smaller patches and modeling the relationships between them through self-attention mechanisms. What this self-attention mechanism does is largely capture the long-range dependencies and general global contextual relations rather than only capturing local spatial patterns. These projection matrices, Equations 10, 11, 12, respectively $W^{Q}$ , $W^{K}$ , $W^{V}$ , respectively, $d_{k}$ is the dimensionality of the key vectors, Q and $K^{T}$ by dot products will get the attention scores. Further, these attention scores are normalized using the softmax function as described in Equation 9.


(9) $$Attention\left(Q,K,V\right)=softmax\left(\frac{QK^{T}}{\sqrt{d_{k}}}\right)V$$

(10) $$Q=SW^{Q}$$

(11) $$K=SW^{K}$$

(12) $$V=SW^{V}$$

The dot product between the query and key is taken to determine how much attention should be given to the different regions of the image by allowing LVMs to emphasize relevant spatial relationships that are significant in making the distinction between malignant and benign cervical MRI and CT images. To increase feature diversity, LVM implements multi-head self-attention, whereby several attention functions are applied in parallel according to Equation 13, where $W^{O}\;$ is the output-projection matrix, and h is the number of attention heads as shown in Equation 14.


(13) $$MHSA\left(Q,K,V\right)=Concat\left(head_{1},\;head_{2},\;head_{3},\;\ldots head_{h}\right)W^{O}$$

(14) $$head_{i}=Attention\left(QW_{i}^{Q},KW_{i}^{K},VW_{i}^{V}\right)$$

Multi-headed attentions whereby LVMs can pick up several relationships in the image instantaneously, hence enhancing the chances of classifying it correctly. For instance, one head can be used to identify the tumor margins while the other investigates the texture variations indicating malignancy. Position encoding becomes necessary in vision transformers since transformers do not hold an embedded spatial hierarchy like CNN architecture; some position encoding is needed for retaining spatial information. The position encoding function is usually defined as shown in Equations 15, 16:


(15) $$PE_{\left(pos.2i\right)}=\sin\left(\frac{pos}{10000^{2i/d}}\right)$$

(16) $$PE_{\left(pos.2i+1\right)}=\cos\left(\frac{pos}{10000^{2i/d}}\right)$$

Here, pos denotes the position in the sequence, d indicates the embedding dimension, while i represents the feature index. The position encodings are added to the input image patches before passing them through the transformer layers to retain spatial information.

### 2.4 InternImage

InternImage is a hybrid model that blends the efficiency of the CNNs with the flexibility that ViTs afford (22). We built an InceptionV3-based model through which we brought in multiscale feature extraction combined with dynamic convolutions and self-attention to improve performance. This proposed model brings forth a detailed mathematical explanation of InternImage with InceptionV3 as the backbone model. InternImage comprises the following major components. InceptionV3, the base feature extractor, extracts low and middle-level features through Inception modules, processing images over multiple scales (23). Dynamic Convolutional Blocks replace static convolution filters with input-dependent kernels for enhanced adaptability. Self-Attention Mechanism Captures long-range dependencies for better feature representation. Hierarchical Representation Learning: pyramidal feature extraction at several levels for image processing. Fully Connected Layers for Final Classification Prediction Layer with Softmax Activation.

**Figure 4:**
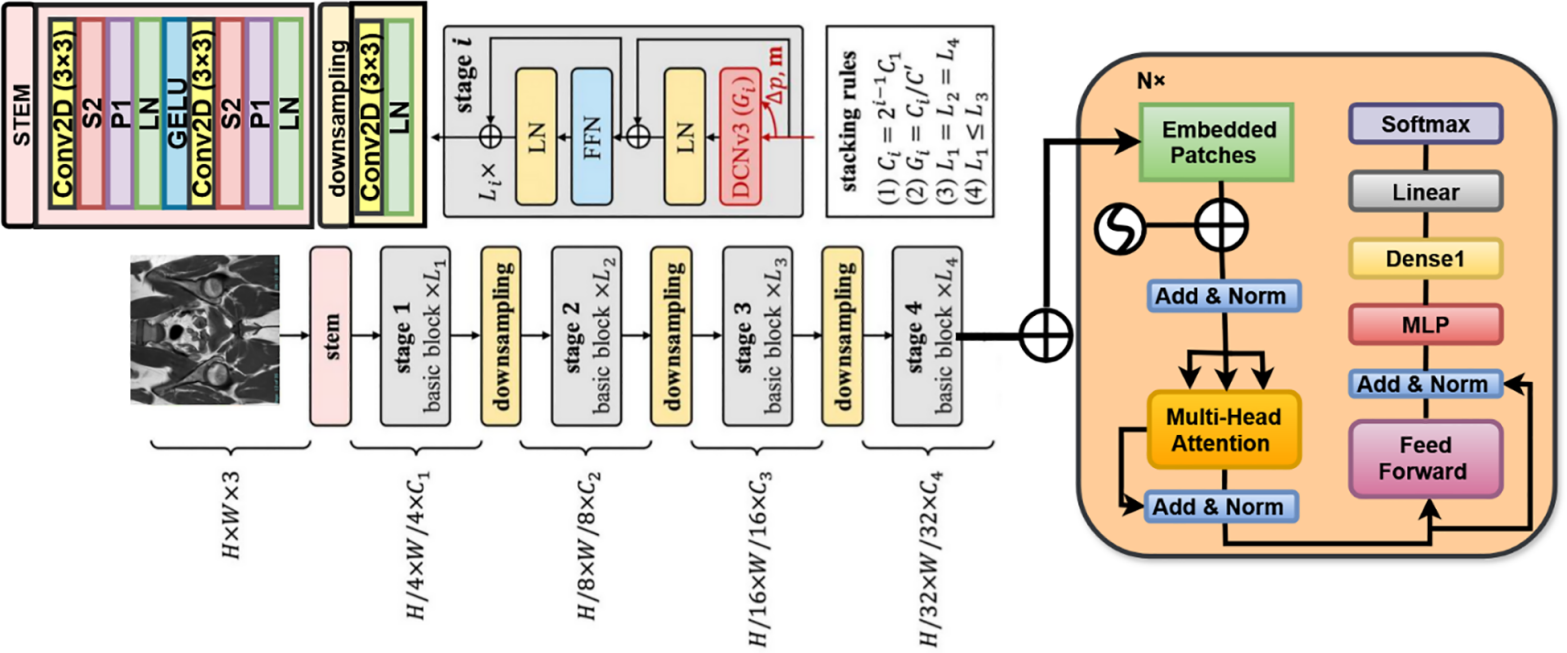
Architecture design of the internimage.

Multi-scale feature extraction, in principle, takes place with different convolutional filters, in parallel, having diverse sizes of kernel (1×1, 3×3, 5×5) . This process captures spatial features at different scales, which makes it robust for the detection of heterogeneous structures in medical images (24). Instead of making use of the standard 3×3 convolution, InceptionV3 uses 2 consecutive convolutions of 1 and 1×3 to factorize the same. This helps to reduce the number of parameters and the computation cost. In this paper, an additional classifier has been integrated into the intermediate layer so that it can improve the flow of gradients during training and mitigate the chances of vanishing gradients in deep networks. Normalizes the feature maps to stabilize and accelerate the training. **Figure 4** Illustrates the working architecture of the model.

#### 2.4.1 Mathematical formulation of inception modules

The mathematical formulation of an Inception module consists of multiple convolutional layers operating in parallel, as shown in Equations 17–21:


(17) $$F_{1\times 1}=\sigma \left(W_{1\times 1}*S+b_{1\times 1}\right)$$

(18) $$F_{3\times 3}=\sigma \left(W_{3\times 3}*S+b_{3\times 3}\right)$$

(19) $$F_{5\times 5}=\sigma \left(W_{5\times 5}*S+b_{5\times 5}\right)$$

(20) $$F_{pool}=\sigma \left(W_{pool}*S+b_{pool}\right)$$

(21) $$F_{concat}=\left[F_{1\times 1},F_{3\times 3},F_{5\times 5},F_{pool}\right]$$

Where S is an input feature map. $F_{k\times k},b_{k\times k}$ is weights and biases for convolutional layers. σ is an activation function (e.g., ReLU). $F_{concat}$ is a concatenated feature representation from different filters. This structure ensures that both fine-grained details and large-scale structures are captured effectively.

#### 2.4.2 Dynamic convolution mechanism in InternImage

Traditional CNNs use static convolutional filters that remain unchanged once trained (22). However, InternImage introduces dynamic convolutions, where filters adapt based on the input. A CNN convolution operation is defined by Equation 22, Dynamic convolution in InternImage. Instead of using fixed kernels, InternImage generates convolutional filters dynamically using by Equations 23, 24.


(22) $$F_{l}=\sigma \left(W_{l}*S+b_{l}\right)$$

(23) $$W_{l}=g\left(S\right)\cdot W_{base}$$

(24) $$F_{1\times 1}=\sigma \left(\left(g\left(S\right)\cdot W_{base}\right)*S+b_{l}\right)$$

Here, $F_{l}$ depicts the feature map at the layer. l S is the input feature map. $W_{l}$ , $b_{l}$ convolution filters, and biases. σ is an activation function (e.g., ReLU). $W_{base}$ . It is the base convolutional kernel. The learnable function g(S) generates a new kernel conditioned on the input.

CNNs use local receptive fields, whereas InternImage establishes long-range dependencies based on the self-attention mechanism, inspired by Vision Transformers. The mathematical formulation of self-attention is computed using query (Q), key (K), and value (V) matrices by Equation 9. InternImage extracts features through multiple levels of image processing, like Feature Pyramid Networks (FPNs). During the hierarchical feature-extraction phases, Low-Level Features (Shallow CNN Layers) are obtained in the early convolutional layers to capture basic textures and edges.

### 2.5 Shark optimization algorithm

Shark Optimization Algorithm (SOA) is a kind of swarm intelligence-based method that imitates sharks in foraging and predatory behavior in the ocean (25). Sharks maintain a balance between exploration and exploitation so that prey can be tracked and sufficiently captured. In SOA, this is all mathematically modeled to handle some complex optimization problems (26).

The algorithm iteratively works. A population of candidate solutions (sharks) evolves toward the optimal solution.

Each shark is a candidate solution randomly initialized in the search space. Where $S_{i}\;$ position of the $i^{th}\;$ shark. $S_{min}$ , $S_{max}\;$ are the lower and upper bounds of the search space, as shown in Equation 25: and r is a random number drawn from the uniform distribution [0,1].


(25) $$S_{i}\;=S_{min}+r\times \left(S_{max}-S_{min}\right)$$

Each shark evaluates its fitness based on the objective function $f\left(S_{i}\right)$ , which depends on the optimization problem. The shark with the best fitness is recorded as the current best solution $S^{*}$ . For a minimization problem by the Equation 26, or for a maximization problem by the Equation 27.


(26) $$S^{*}=\arg minf\left(S_{i}\right)$$

(27) $$S^{*}=\arg maxf\left(S_{i}\right)$$

Movement strategy sharks update their position based on two key components. Cognitive component sharks remember their best-known position. The social component sharks adjust their movement based on the best-performing shark. As shown in Equations 28, 29:


(28) $$V_{i}^{\left(t+1\right)}=V_{i}^{\left(t\right)}+C_{1}r_{1}\left(S^{*}-S_{i}^{\left(t\right)}\right)+C_{2}r_{2}\left(S_{best}-S_{i}^{\left(t\right)}\right)$$

(29) $$V_{i}^{\left(t+1\right)}=S_{i}^{\left(t\right)}+S_{i}^{\left(t+1\right)}$$

Where $V_{i}^{\left(t\right)}$ is the velocity of the $i^{th}\;$ shark at iteration t . $C_{1},C_{2}$ are acceleration coefficients controlling cognitive and social learning (27). $r_{1},r_{2}$ are random numbers in [0,1]. $S_{best}\;$ is the position of the current best-performing shark. $S_{i}^{\left(t\right)}$ is the current position of the shark. $S^{*}\;$ is the best position found globally so far.

Exploration and exploitation balance sharks alternating between searching for new areas (exploration) and refining the best-known solution (exploitation). A control factor α is introduced to balance these phases, α  gradually decreases to encourage more exploitation in later iterations as shown in Equation 30:


(30) $$S_{i}^{\left(t+1\right)}=S_{i}^{\left(t\right)}+\alpha V_{i}^{\left(t+1\right)}$$

Adaptive behavior and convergence to avoid getting stuck in local optima, the velocity update includes an adaptive random movement term as shown in Equation 31:


(31) $$V_{i}^{\left(t+1\right)}=\beta V_{i}^{\left(t\right)}+\left(1-\beta \right)\left(S^{*}-S_{i}^{\left(t\right)}\right)+\gamma \times randn()$$

**Figure 5:**
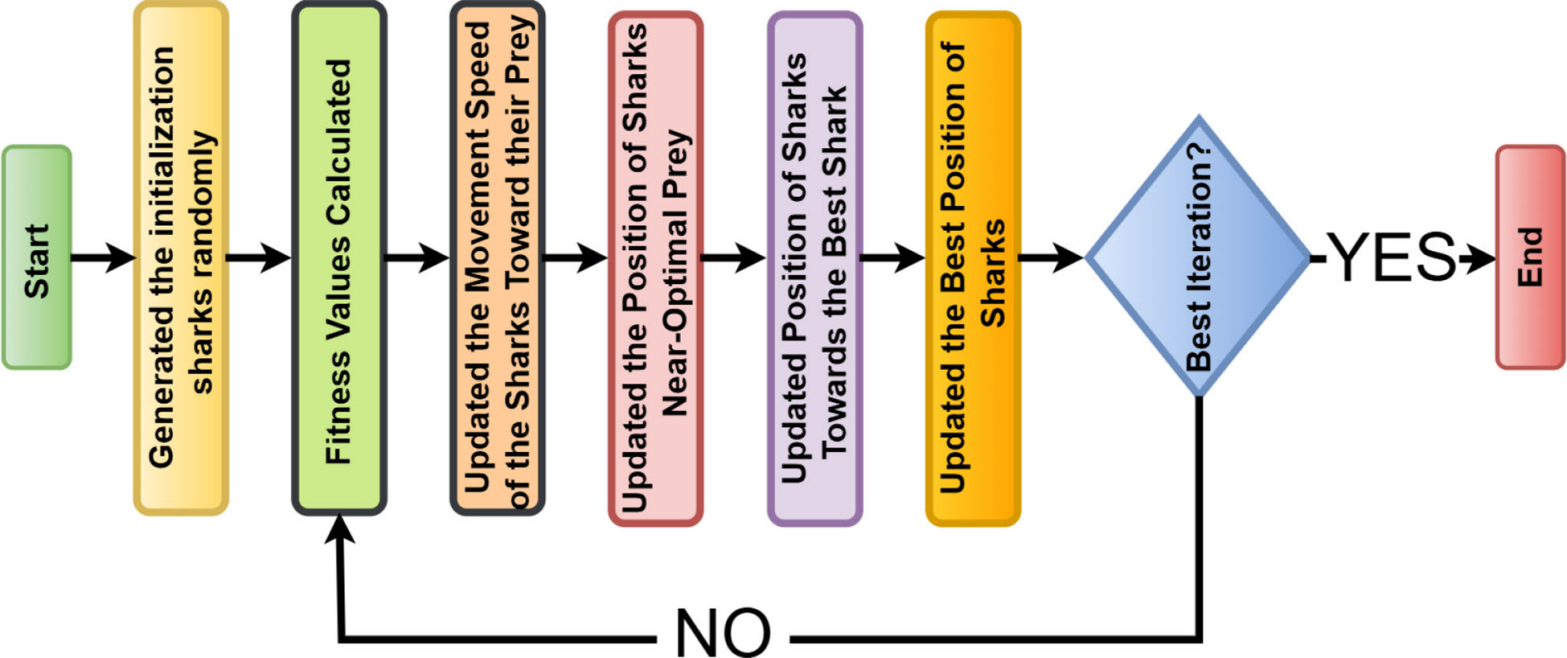
A flow graph for the shark optimization algorithm (SOA).

Where β is a momentum coefficient (typically 0.5–0.9). γ controls the strength of random movement (higher values encourage more exploration). randn()  is a normally distributed random number. The algorithm runs for a fixed number of iterations, stopping when no significant improvement is observed. **Figure 5** illustrates the Bayesian optimization flowchart.

### 2.6 Build of proposed model

The proposed model is an advanced ensemble deep learning framework for the classification of cervical MRI and CT images into the applicable categories of Benign or Malignant, or Normal. The model tries to integrate the pros of two deep learning architectures (28). LVM-A custom convolutional neural network (CNN) that extracts deep spatial and structural features from cervical medical images. InternImage (An InceptionV3-based Model)-A pre-trained feature-extraction model to level up classification performance. The ensemble obtains optimal accuracy in the classification using the Shark Optimization Algorithm (SOA) for determining the best weight coefficients (29).

To enhance the diagnostic accuracy of cervical cancer classification, this study proposes a novel hybrid framework that combines the strengths of the Large Vision Model (LVM), InternImage, and the Shark Optimization Algorithm (SOA). The proposed architecture introduces a unique synergy between spatial and semantic feature representations.

InternImage, built upon the InceptionV3 backbone, is utilized to extract deep pre-trained features that are highly sensitive to tumor-specific patterns, which are particularly useful in distinguishing benign from malignant cases. In contrast, the Large Vision Model (LVM) contributes fine-grained spatial features, enabling the detection of early pathological changes in cervical tissue morphology.

To effectively integrate the outputs of both models, we adopt the Shark Optimization Algorithm (SOA) as a dynamic weighting mechanism. SOA simulates the intelligent hunting behavior of sharks, wherein it explores the solution space adaptively to optimize the weight parameters assigned to each model’s output. Unlike traditional fusion techniques such as fixed-weight averaging or majority voting, SOA dynamically adjusts these weights based on performance feedback during training. This allows the model to avoid overfitting and improves generalization across datasets with different imaging modalities (CT and MRI).

The advantage of using SOA lies in its adaptive learning capability, which enables the system to better respond to variations in data quality, imaging characteristics, and class distributions. This dynamic fusion process significantly reduces misclassification, especially in challenging borderline cases, and outperforms conventional static ensemble approaches.

#### 2.6.1 Individual models and their mathematical formulation

The LVM comprises several convolutional layers and max-pooling operations, which are then followed by fully connected layers, all designed to extract features from cervical MRI and CT images (21). Mathematically, forward propagation in LVM is defined as shown in Equation 32:


(32) $$F_{LVM}\left(S\right)=Softmax\left(W_{3}\cdot \sigma \left(W_{2}\cdot \sigma \left(W_{1}\cdot S+b_{1}\right)+b_{2}\right)+b_{3}\right)$$

Where S is the cervical MRI or CT input image. The different weight matrices for the different layers are $W_{1}$ , $W_{2}$ , $W_{3}$ . $b_{1}$ , $b_{2}$ $,b_{3}$ are the bias terms. σ is the activation function (ReLU) (30). The score is generated through Softmax over three classes: Benign, Malignant, and Normal. The very purpose of the LVM is to capture minute anatomical differences as a way of understanding malignant and benign tumors in cervical cancer diagnosis. InternImage is built on InceptionV3, which is a pre-trained model serving as a feature extractor (31). The last classification layers are fine-tuned to classify cervical MRI and CT images, as shown in Equation 33:


(33) $$F_{\text{InternImage }}\left(S\right)=Softmax\left(W_{dense}\cdot G\left(S\right)+b_{dense}\right)$$

G(S) denotes deep feature embeddings extracted via InceptionV3. The variables $W_{dense}\;and\;b_{dense}$ denote the last layer’s weight matrix and bias, respectively. The Softmax function assigns probabilities to the three classes: Benign, Malignant, and Normal. InternImage retains robust generalization using pre-trained deep features to reduce overfitting on small medical datasets.

#### 2.6.2 Ensemble learning with shark optimization algorithm

Instead of considering a model in isolation, the ensemble method improves classification accuracy by aggregating predictions from various architectures (32). The ensemble decision function is Equation 34. Where $P_{LVM},P_{\text{InternImage }}$ . With probability outputs from LVM and InternImage. $\omega_{1},\omega_{2}$ are optimal ensemble weights. These weights are determined using the Shark Optimization Algorithm (SOA) to ensure model fusion for the classification of cervical cancer.


(34) $$P_{ensemble\;}=\omega_{1}P_{LVM}+\omega_{2}P_{\text{InternImage }}$$

#### 2.6.3 Shark optimization algorithm for weight optimization

SOA optimizes the weights using a given fitness function (33). Where ${\hat{Y}}_{i}$ is max ( $P_{ensemble\;},i$ ) meaning the predicted class. $Y_{i}$ is the true class label (Benign, Malignant, or Normal). $\delta \left({\hat{Y}}_{i},Y_{i}\right)$ =1 if the prediction is correct, otherwise 0. N is the total cervical MRI and CT samples. SOA updates the ensemble iteratively for maximum classification accuracy, as shown in Equation 35:


(35) $$Fitness\left(\omega_{1},\omega_{2}\right)=\frac{1}{N}\sum_{i=1}^{N}\delta \left({\hat{Y}}_{i},Y_{i}\right)$$

LVM and InternImage will carry out cervical MRI and CT classification. Optimized ensemble learning by SOA increases the accuracy and generalization. Successfully isolates Benign from Malignant and Normal cases. Facilitates early detection of cervical cancer, which allows for timely medical intervention.

Hyperparameter tuning was conducted to optimize the performance of the developed deep learning model (34). The values selected were optimal on systematic experimentation, using the Shark Optimization Algorithm (SOA) in **Table 2**. The learning rate of 0.001 turned out to be optimal for training to have a stable and efficient model development environment. Batched 32 achieved good speed in the computation as well as convergence of the model. Adam, of all the optimizer functional forms tested, produced the highest performance. The dropout rate for overfitting was set at 0.3, having a very general model. The model’s robustness further increased with L2 regularization of 0.0005. On training with the model for 50 epochs, learning was sufficiently done not to require intense computation. A hidden layer size of 256 turned out to be quite productive.

**Table 2:** Hyperparameter optimization results using the shark optimization algorithm.

Hyperparameter	Value range	Best value
Learning Rate	0.0001 - 0.01	0.001
Batch Size	8 - 64	32
Optimizer	Adam, SGD, RMSprop	Adam
Dropout Rate	0.2 - 0.5	0.3
L2 Regularization	0.0001 - 0.01	0.0005
Number of Epochs	10 - 100	50
Hidden Layer Size	64 - 512	256
Gradient Clipping	0.1 - 5.0	1.0

while controlling an exploding gradient with a gradient clipping value of 1.0, which helped stabilize the training process. Such hyperparameters worked well for this model to get high validation accuracy.

To ensure a fair comparison between the proposed model and baseline architectures, all models underwent a dedicated hyperparameter optimization process. For the proposed model, the Shark Optimization Algorithm (SOA) was used to dynamically optimize parameters such as learning rate, dropout rate, and model fusion weights. For the baseline models, a grid search approach was applied to determine optimal values for key parameters, including learning rate, number of hidden units, and regularization strength. All models were trained and validated using the same 5-fold cross-validation strategy.

### 2.7 Methods of transfer learning

**Figure 6:**
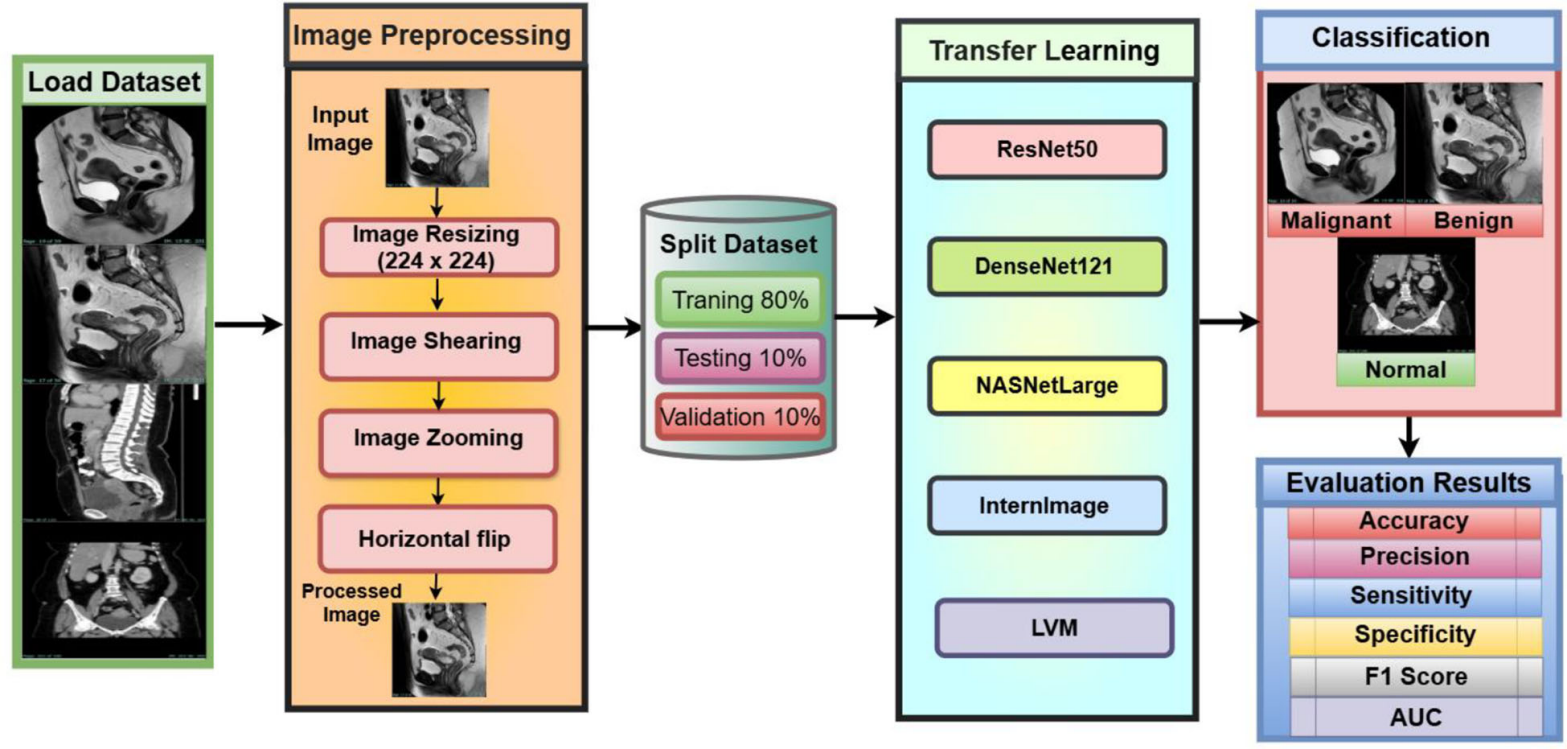
Working methods of transfer learning.

Transfer learning is an excellent technique under deep learning, where the model or with pre-trained parameters to help improve the performance of the model on a new dataset (35). Rather than developing a deep neural architecture from scratch, the model would probably depend on the knowledge gained from a larger dataset scale, such as ImageNet, that improves feature extraction and classification. In this study (36), five advanced deep learning architectures (ResNet50, DenseNet121, NASNetLarge, InternImage, and LVM) were utilized to classify cervical MRI and CT images into three categories: Benign, Malignant, and Normal. As shown in **Figure 6**.

### 2.8 Evaluation proposed method

The evaluation of the cervical cancer classification model uses a standardized set of evaluation metrics to assess the ability of the model to distinguish Benign from Malignant and Normal cases using MRI and CT images (37). The effectiveness of the model is quantified through the metrics outlined below. In simple terms, accuracy considers how many predictions made by the model are correct in general. Precision tells us how many out of those predicted to be positive by the model are positive. Sensitivity, also called Recall or True Positive Rate TPR, indicates the proportion of actual positives that are identified correctly. Specificity evaluates the correct identification of negative cases by the model. The F1 Score, being defined as an inverse relationship between Precision and Sensitivity, balances false positives and false negatives, as shown in Equations 36–40:


(36) $$Accuracy=\frac{TP+TN}{TP+TN+FP+FN}$$

(37) $$\text{Precision}=\frac{TP}{TP+FP}$$

(38) $$\text{Sensitivity }=\frac{TP}{TP+FN}$$

(39) $$\text{Specificity }=\frac{TN}{TN+FP}$$

(40) $$\text{F}1\text{ Score}=2*\frac{\text{Precision}*\text{Sensitivity}}{\text{Precision}+\text{Sensitivity}}$$

The Area Under the Curve (AUC) is a statistical measure evaluated to assess the performance of the model in classification. It represents the probability that a randomly chosen positive case (Benign, Malignant, or Normal) ranks higher than a randomly chosen negative case. The AUC is calculated from the relation between True Positive Rate (TPR) and False Positive Rate (FPR) as shown in Equations 41–43:


(41) $$\text{TPR}=\frac{\text{TP }}{\text{TP}+\text{FN}}$$

(42) $$\text{FPR}=\frac{\text{FP }}{\text{FP}+\text{TN}}$$

(43) $$AUC=\int_{0}^{1}TPRd\left(FPR\right)$$

By using the trapezoidal rule for numerical approximations. Thus, a high AUC value (closer to 1) offers a better ability to discriminate among the three classes: Benign, Malignant, and Normal as shown in Equation 44. Such a kind of evaluation assures the proposed model is true, reliable, and clinically applicable for the classification of cervical MRI and CT images.


(44) $$AUC=\sum_{i=1}^{n-1}\left(FPR_{i+1}-FPR_{i}\right)*\frac{FPR_{i}+FPR_{i+1}}{2}\;$$

## 3 Result analysis

This research compares MRI and CT imaging to make a cervical cancer diagnosis. A hybrid model that integrated with an LVM model from Convolutional Neural Networks (CNNs) and an InternImage model from InceptionV3 was employed to make predictions on its own. The Shark Optimization Algorithm (SOA) was employed to optimally fuse the outputs of the two models and enhance classification performance by finding the optimal weights for each model and classifying cervical MRI and CT scans into normal, malignant, and benign. The King Abdullah University Hospital in Jordan served as the source for the KAUH-CCMD and KAUH-CCTD datasets. The identical set of parameters was used to train each model: 50 epochs, learning rate of 0.001, Adam activation function, and class cross-entropy loss function. For this study, the dataset is split into three subsets: 80% for training, 10% for validation, and 10% for testing. Additionally, the models were trained locally using an RTX 3050 GPU and a Jupyter laptop.

### 3.1 Model performance evaluation and analysis on KAUH-CCTD

**Figure 7:**
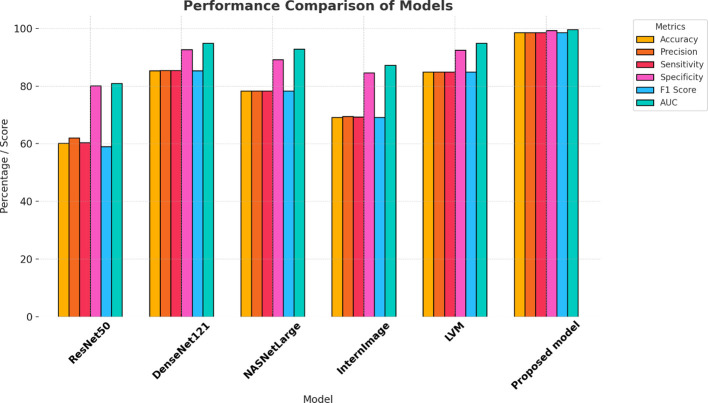
Model performance analysis using KAUH-CCTD.

The proposed model appears to have performed best based on the provided evaluation metrics. The proposed model had the highest precision, AUC, sensitivity, specificity, F1 score, and accuracy compared to all the other models for cervical cancer diagnosis from CT images. Precision represents the ratio of correctly predicted positive cases out of all the predicted positive cases. High accuracy means a low false positive rate in the model, which means it is more accurate to predict positive cases. As can be seen from **Table 3**. The proposed model’s accuracy was 98.49%, specificity was 99.23%, and AUC was 99.54%, proving its efficiency for the diagnosis of cervical cancer using CT images. The LVM and DenseNet121 model accuracy was approximately equal for the CT image classification task, with LVM correctly classifying 84.84% and DenseNet121 correctly classifying 85.35%. The lowest accuracy was recorded by the ResNet50 model in cervical cancer diagnosis, with an accuracy of 60.10%. The result indicates the effectiveness of the suggested model in cervical cancer diagnosis using the CT image. **Figure 7** demonstrates the model’s performance.

**Table 3:** Evaluating the effectiveness of models using the KAUH-CCTD dataset.

Model	Accuracy	Precision	Sensitivity	Specificity	F1 score	AUC
ResNet50	60.10	61.99	60.36	80.11	58.98	80.90
DenseNet121	85.35	85.41	85.37	92.69	85.32	94.82
NASNetLarge	78.28	78.25	78.28	89.14	78.25	92.82
InternImage	69.19	69.51	69.22	84.57	69.19	87.15
LVM	84.84	84.86	84.89	92.43	84.85	94.83
Proposed model	98.49	98.51	98.48	99.23	98.49	99.54

**Figure 8:**
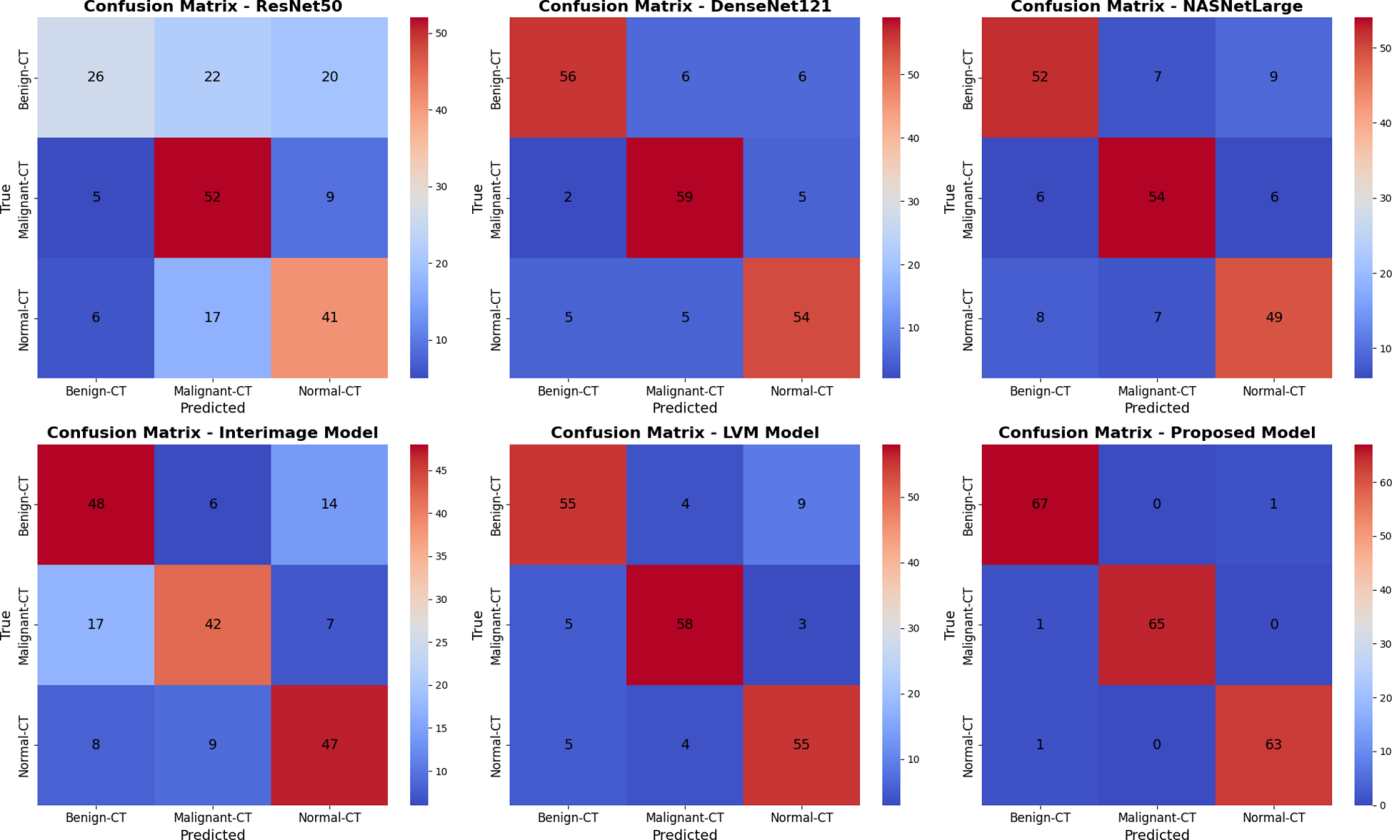
Confusion matrix for all models using KAUH-CCTD.

**Figure 8** shows six confusion matrices for comparing the performance of different deep models, ResNet50, DenseNet121, NASNetLarge, InterImage Model, LVM Model, and the proposed model in classifying cervical CT scans of three classes: benign, malignant, and normal. Each confusion matrix displays the correct and incorrect predictions within these classes. The model suggested has the highest classification rate among cervical CT scans with near-perfect diagonal values (67, 65, 61), which means perfect classification of all three classes with minimal misclassification. DenseNet121 and NASNetLarge perform well in CT image diagnosis. ResNet50 and the InterImage Model experience much confusion among classes, with high misclassification of benign and normal CT scans. The results confirm the superiority of the new model in accurately diagnosing cervical cancer CT images for all classes.

### 3.2 Model performance evaluation and analysis on KAUH-CCMD

**Figure 9:**
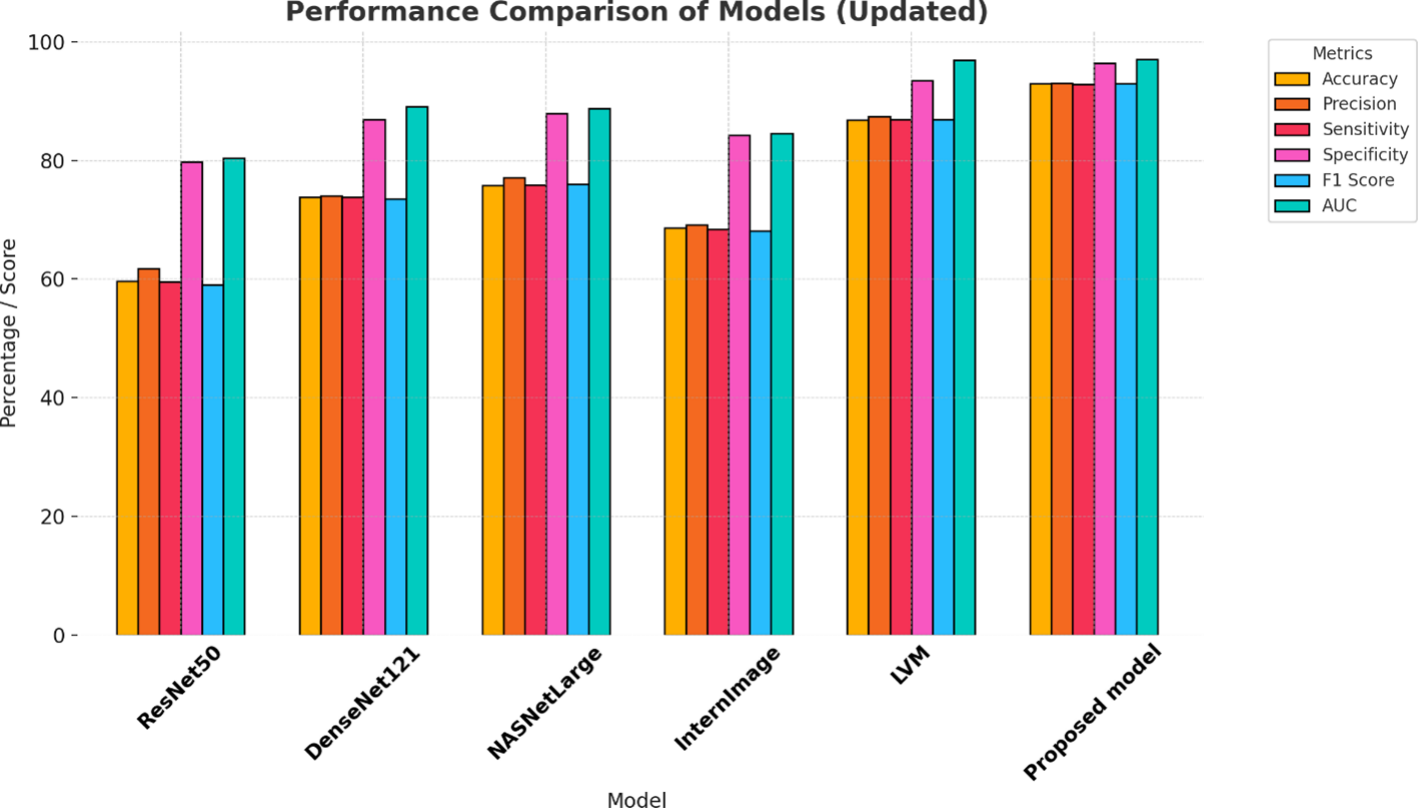
Model performance analysis using KAUH-CCMD.

In this section, the performance of the model was evaluated with the KAUH-CCMD dataset for the diagnosis of cervical cancer from MRI images. When trained, the suggested model gave an impressive accuracy of 92.92%, indicating that it predicted the result correctly for all the test set samples, as shown in **Table 4**. When comparing the performance of the proposed model with other models, it outperformed the others. The specificity was 96.46%, and the AUC was 97.01%, demonstrating the model’s effectiveness in diagnosing cervical cancer on MRI images. LVM ranked second in diagnosing cervical cancer with an accuracy of 86.86%. DenseNet121 and NASNetLarge also performed the same, with the accuracy of 73.79% and 75.75%, respectively. The worst-performing model among the models was ResNet50 with an accuracy of 59.59%. The results demonstrate the effectiveness of the proposed model for cervical cancer diagnosis from MRI. **Figure 9** demonstrates the effectiveness of the model.

**Table 4:** Evaluating the effectiveness of models using the KAUH-CCMD dataset.

Model	Accuracy	Precision	Sensitivity	Specificity	F1 score	AUC
ResNet50	59.59	61.83	59.54	79.75	59.03	80.35
DenseNet121	73.79	74.05	73.79	86.91	73.52	89.06
NASNetLarge	75.75	77.09	75.84	87.94	76.02	88.72
InternImage	68.68	69.16	68.46	84.30	68.14	84.59
LVM	86.86	87.47	86.96	93.47	86.93	96.94
Proposed model	92.92	93.09	92.87	96.46	92.93	97.01

**Figure 10:**
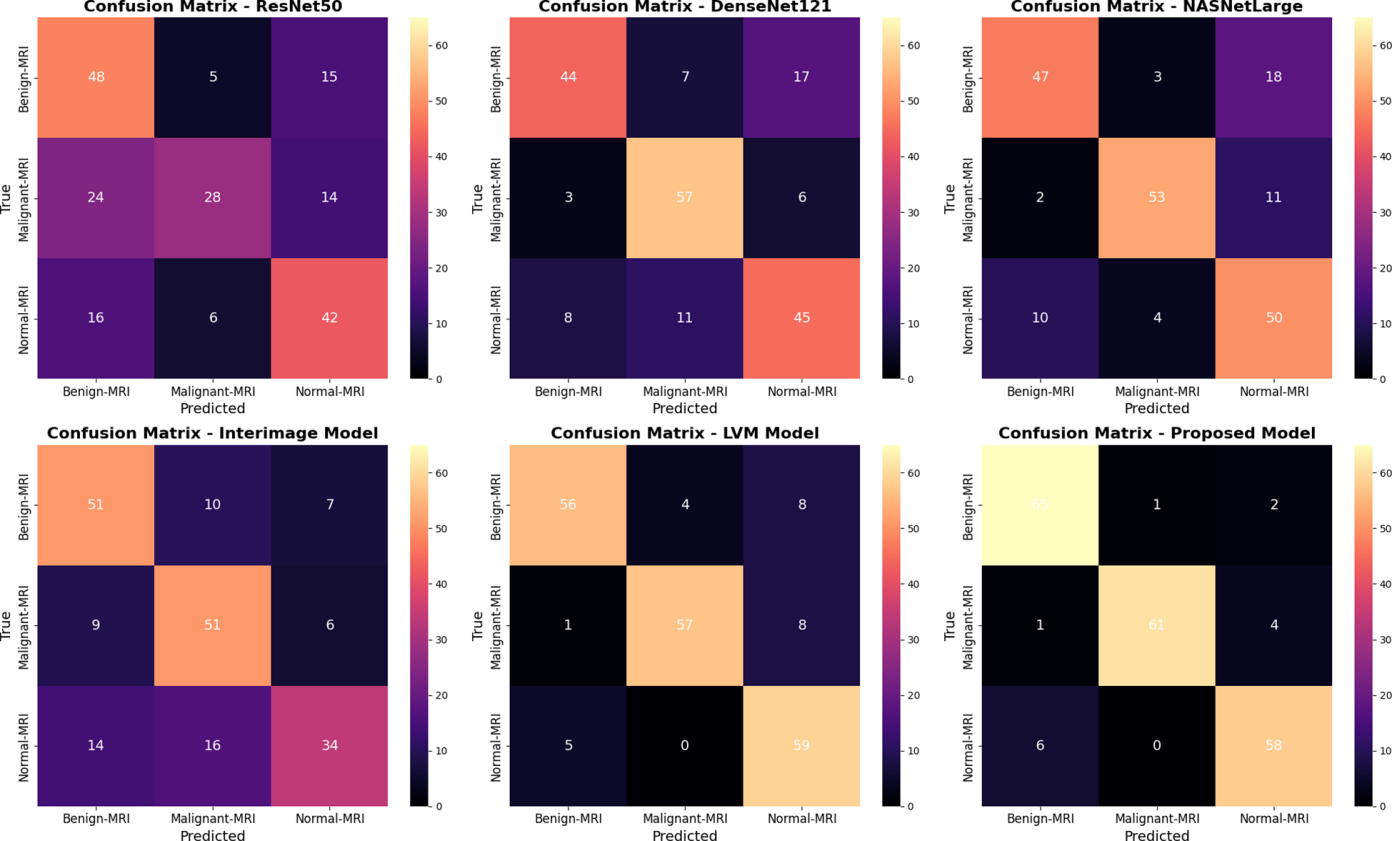
Confusion matrix for all models using KAUH-CCMD.

**Figure 10** shows six confusion matrices to analyze the performance of different deep learning models (ResNet50, DenseNet121, NASNetLarge, InterImage, LVM, and Proposed Model) to classify MRI scans into three classes: Benign-MRI, Malignant-MRI, and Normal-MRI. Each matrix illustrates the correct and wrong predictions for each class in a graphical format, with the best predictions on the diagonal. The Proposed Model stands out clearly with high accuracy and low misclassifications as indicated by its high diagonal values (65, 61, 60). The LVM Model is also satisfactory, with somewhat more errors. ResNet50 and DenseNet121, on the contrary, show high confusion, particularly between malignant and other classes, indicating lower discriminatory power. The NASNetLarge and InterImage models provide moderate performance, better than ResNet50 but still lower compared to the Proposed and LVM models. Overall, the Proposed Model is seen to provide improved classification for MRI-based diagnosis in all classes.

### 3.3 Comparison of MRI and CT in the diagnosis of cervical cancer using the proposed model

**Figure 11:**
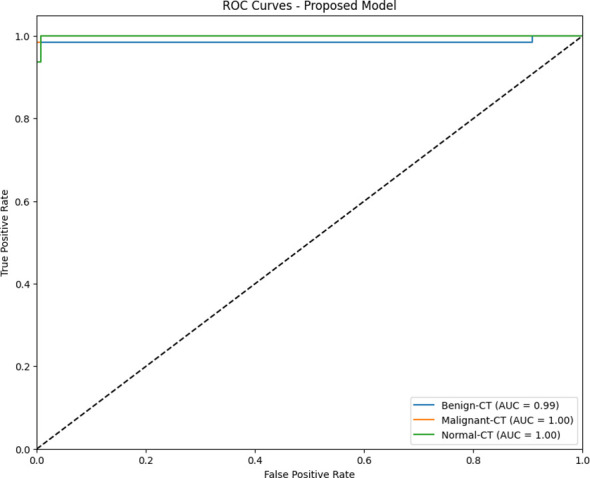
ROC curves for the classification of cervical cancer using KAUH-CCTD.

The main objective of the present study is to analyze the ability of the suggested model for the proper diagnosis of cervical cancer between CT scan and MRI. According to the comparison of KAUH-CCMD and KAUH-CCTD datasets, the suggested model is more precise in diagnosing cervical cancer in CT than MRI, with an accuracy of 98.49% and an area under the curve (AUC) of 99.54%. **Figure 11** shows the ROC plots of the true positive rate (TPR) for each class versus the false positive rate (FPR). The AUC values indicate good performance in classification in that both benign and malignant possess 0.99 and 1.00 accuracy, respectively. A random classifier is depicted as a dashed diagonal line; the better the model performs, the closer the curves are to the upper left corner.

**Figure 12:**
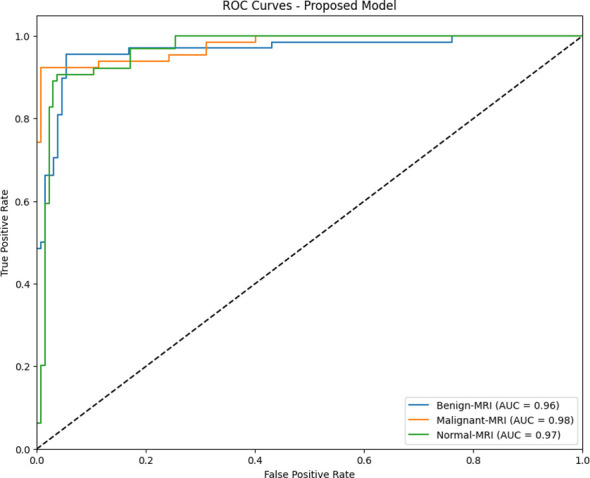
ROC curves for the classification of cervical cancer using KAUH-CCMD.

The model provided a diagnosis accuracy of 92.92% and an area under the curve (AUC) of 97.01% for MRI-based cervical cancer diagnosis. The AUC values were 0.96 for benign, 0.98 for malignant, and 0.97 for normal, as reflected in **Figure 12**.

The proposed model demonstrated a higher diagnostic accuracy on CT images (98.49%) compared to MRI images (92.92%). This performance gap can be attributed to several factors. First, CT images generally provide higher spatial resolution and more consistent contrast levels, which facilitates more precise feature extraction by the model. In contrast, MRI scans are prone to variability due to differences in scanning protocols, magnetic field strength, and susceptibility to noise and artifacts, which can hinder deep feature learning.

Second, specific pathological features of cervical cancer, such as tumor boundaries and calcifications, tend to be more clearly visible in CT scans. These distinct patterns enhance the model’s ability to differentiate between benign, malignant, and normal tissues. Finally, the current hybrid architecture, which leverages spatially focused features (LVM) and deep semantic features (InternImage), may be more compatible with the structural consistency present in CT images. These factors combined contribute to the observed difference in modality-specific classification accuracy.

### 3.4 Statistical analysis

To assess the consistency and comparative strength of the evaluated models, we conducted a statistical analysis based on their accuracy scores using the KAUH-CCTD (CT) and KAUH-CCMD (MRI) datasets. **Table 5** and **Table 6** present the classification accuracy of each model alongside the performance deviation from the best-performing proposed model, and their respective 95% confidence intervals (CI).

**Table 5:** Accuracy, 95% confidence intervals, and deviations – CT Dataset (KAUH-CCTD).

Model	Accuracy (%)	95% CI (±)	Deviation from proposed (%)
Proposed Model	98.49	± 0.45	0.00
LVM	84.84	± 1.22	-13.65
DenseNet121	85.35	± 1.30	-13.14
NASNetLarge	78.28	± 1.70	-20.21
InternImage	69.19	± 2.45	-29.30
ResNet50	60.10	± 3.12	-38.39

**Table 6:** Accuracy, 95% confidence intervals, and deviations – MRI Dataset (KAUH-CCMD).

Model	Accuracy (%)	95% CI (±)	Deviation from proposed (%)
Proposed Model	92.92	± 0.67	0.00
LVM	86.86	± 1.15	-6.06
NASNetLarge	75.75	± 1.72	-17.17
DenseNet121	73.79	± 2.05	-19.13
InternImage	68.68	± 2.47	-24.24
ResNet50	59.59	± 2.96	-33.33

On the CT dataset, the proposed model achieved the highest accuracy of 98.49%, with a narrow 95% CI indicating high reliability and minimal variation across folds. Models such as DenseNet121 and LVM also demonstrated strong performance, yet showed a performance gap of 13.14% and 13.65%, respectively, compared to the proposed model. The lowest-performing model, ResNet50, had a significantly wider CI and a performance drop of 38.39%, indicating higher variability and lower consistency.

Similarly, on the MRI dataset, the proposed model reached an accuracy of 92.92%, with a narrow CI. The LVM model followed at 86.86%, within 6.06% of the proposed model, while ResNet50 again showed the lowest performance, trailing by 33.33%.

Overall, the results reveal a direct relationship between higher accuracy and narrower confidence intervals, suggesting that more accurate models tend to deliver more stable and reliable performance. The proposed model consistently outperformed all other baselines across both datasets, both in absolute performance and statistical stability.

### 3.5 Study limitations

This study has several limitations that should be considered when interpreting the results. First, the retrospective nature of the data collection introduces potential selection bias, which may affect model performance. Second, the use of data from a single institution may limit generalizability to other clinical settings. Third, despite efforts to balance the dataset, some variability in image quality, acquisition protocols, and potential class imbalance remain. Additionally, while the proposed model performs well in classification, it does not yet support tumor localization, staging, or uncertainty estimation. These aspects represent important directions for future development. There are certain limitations to follow when data collection is conducted in hospitals. Legal obligations and patient confidentiality result in stripping off identifying information, making a public statement of clear intent for the study, and protecting data from inappropriate individuals. Proper authority and ethics approval must be sought. Adherence to data minimization policies is essential in that only necessary information is retained and kept for a given period before they are disposed of. Although the dataset used in this study was collected from a single hospital and includes a total of 1,974 images per modality, several data augmentation and validation techniques were applied to mitigate the risks of overfitting and enhance model generalization. Nevertheless, future work will focus on validating the model using multi-center datasets from diverse imaging environments to further assess its robustness and clinical applicability. One of the significant weaknesses of AI systems in healthcare involves a lack of transparency that detracts from reliability and interpretability. Interpretable AI techniques need to be employed to remove this deficiency.

Although the model achieved high classification accuracy, particularly for CT images, the possibility of overfitting remains a valid concern due to the relatively small dataset size and the complexity of the hybrid architecture. To mitigate this, several precautions were implemented, regularization, data augmentation, and early stopping. However, further external validation on independent, multi-center datasets is necessary to fully assess the model’s generalizability and confirm its robustness in broader clinical settings.

## 4 Discussion

Magnetic resonance imaging (MRI) and computed tomography (CT) have facilitated the identification and classification of cervical tumors. Numerous approaches have been explored, including model-based deep learning approaches, radiation-based approaches, and hybrid systems for grading multimodal images. While studies demonstrate the advantage of CT in the detection of endometrial cancer, they also indicate limited datasets, heterogeneity in imaging protocols, and the absence of external validation.

While previous hybrid models (38) have demonstrated strong results on tasks such as colorectal (39) and skin cancer (40) classification, they often relied on fixed architectural blocks or overlooked the limitations of image variability and dataset generalizability. Unlike (41), which focuses solely on cervical cancer with static transformer layers. Moreover, we address the challenge of interpretability by incorporating Grad-CAM visualizations and statistical significance testing to support our claims (42). Thus, this study fills a critical gap by offering a robust, interpretable, and clinically deployable solution for skin lesion classification.

The contributions made by different strategies for improving tumor classification and diagnostic accuracy are discussed below.

### 4.1 Literature review on computed tomography in the diagnosis of cervical cancer

They proposed in a study (43) to develop a multimodal deep-learning model to predict lymph node metastasis (LNM) in cervical cancer from a collection of 233 contrast-enhanced multiphase CT images. Their model blended a three-dimensional MedicalNet pre-trained model for feature extraction and employed the least absolute shrinkage and selection operator (LASSO) regression for feature selection. The model was 88% accurate, with an AUC of 82%, a sensitivity of 83%, and a specificity of 89%. Even though the model was robust, some limitations to the studies were few, including their retrospective nature, potential biases from the collection of data at one center, and external validation required from a larger multi-center dataset for the determination of its generalizability.

A study (44) proposed a deep learning method of automatic segmentation of interstitial needles from post-operative cervical cancer brachytherapy using a database of 70 three-dimensional CT scans. Their model was trained on the detection of metal needles and was evaluated in terms of geometric accuracy metrics with Dice similarity coefficients (DSC) of 88%, 89%, and 90% for three needles. The method demonstrated high accuracy in needle positioning with little dosimetry difference from manual reconstruction. However, the study was constrained by limited dataset size, lack of external validation across modalities, and possible generalizability issues on account of the single-institutional dataset.

A study (45) proposed an artificial neural network (ANN) model for the identification of cervical abnormality from computed tomography (CT) images. Their study employed a dataset of 212 CT images, of which 106 were normal and 106 were abnormal cervical images, sampled from three hospitals. The techniques employed included preprocessing, segmentation by using a region-based snake model, and feature extraction by using a gray-level co-occurrence matrix (GLCM). ANN was then used for classification with a support vector machine (SVM) as the control. ANN was 95.75% accurate as opposed to 92.9% for SVM. While its accuracy was extremely high, the study was hampered by having a limited diversity dataset because cervical CT images were not very large in number, and variations in cancer staging were not extensively tested.

The study (46) proposed a machine learning-based model for predicting the occurrence of malignant cells in pelvic lymph nodes-pelvic lymph node metastasis (PLNM), in the early stages of cervical cancer. It used 832 preoperative computed tomography (CT) scans of patients as a basis for the study. Seven machine learning models, such as logistic regression, random forest, and support vector machine, were compared. Accuracy between the models ranged from 89.1% to 90.6%, sensitivity ranged from 77.4% to 82.4%, and specificities ranged from 92.1% to 94.3%. The study was limited: relatively small dataset size, exclusion of patients who did not undergo CT scans would introduce selection bias, and CT results were not centrally read by radiologists.

### 4.2 Literature review on magnetic resonance imaging in the diagnosis of cervical cancer

In a study by Qin (47), deep multiple-instance learning (D-MIL) was employed to predict lymph node metastasis (LNM) in operable cervical cancer patients using MRI data from a cohort of 392 patients. The model used for imaging feature extraction with no manual tumor annotation was based on ResNet-50, which achieved AUC scores of 75.7%, 71.4%, and 76.5% for the training, internal, and external cohorts, respectively. The introduction of clinical parameters led to a hybrid model (M3) attaining AUC scores of 83.8, 76.4, and 83.5. The study was limited mainly due to its retrospective nature and small sample size, which might have introduced bias.

The study (48) investigated the detection of cervical cancers using a dataset that comprises 900 cancerous and 200 non-cancerous MRI images. Four machine-learning models have been applied for image classification, namely VGG16, CNN, KNN, and RNN. Additionally, robust preprocessing techniques, including standardization, normalization, and noise filtering, have been employed to enhance the dataset’s quality. The best-performing model was VGG16, with an accuracy of 95.44%. The accuracies of CNN, KNN, and RNN were 92.3%, 89.99%, and 86.23%, respectively. Although VGG16 achieved good accuracy, according to the authors, factors limiting its performance included dataset imbalance, dependency on pre-trained models, and variations in MRI acquisition settings.

In the study (49), MRI is considered the gold standard for local staging in cervical cancer, as it offers better soft tissue contrast and assesses tumor size, the extent of stromal infiltration, and pelvic lymph node involvement. The study revealed that a high-resolution T2-weighted MRI had an accuracy of 88% and a negative predictive value of 94-95% for detecting parametrial invasion. It also demonstrated that DWI-MRI improved sensitivity and specificity to 86% and 84%, respectively, for lymph node metastasis diagnosis. Its limitation is high cost, longer scanning time, and reduced accuracy in the evaluation of retroperitoneal disease. The study also argued that MRI, together with imaging modalities such as PET-CT, would improve overall diagnostic yield as well as for treatment planning.

The study by (50) aimed at predicting the response of patients having locally advanced cervical cancer (LACC) to chemoradiotherapy (CRT). This research was based on a dataset comprising 252 subjects who underwent pre-treatment MRI scans. Two models were created: a handcrafted radiomics (HCR) model that involved feature extraction of 1,890 imaging features and adopted the use of an SVM classifier, and a deep learning radiomics (DLR) model that introduced the use of a 3D convolutional neural network for the same purposes. The model DLR scored higher than HCR, with an accuracy of 73.2% compared to the latter’s 59.8%. For clinical factors, integrated accuracy was raised to 77.7% for DLR and 67.6% for HCR. Limitations included the sample size and the lack of external validation, which affected the study’s generalizability.

The research work conducted in (51) It is about the detection of cervical cancer using a multiparametric MRI dataset containing 177 images. The purpose of this research was to create a radionics-based model capable of predicting lymph-vascular space invasion (LVSI). From T2-weighted MRI (T2WI), diffusion-weighted imaging (DWI), and dynamic contrast-enhanced T1-weighted imaging (DCE T1WI), the techniques of maximum relevance and minimum redundancy (mRMR) and LASSO regression were used to select thirteen significant features. The resultant area under the curve (AUC) was found to be equal to 83.8% in the training cohort and equal to 83.7% in the testing cohort for this radiomics nomogram, with 78.0% and 72.2% accuracy in the respective cohorts.

An experiment by (52) utilized convolutional neural networks (CNNs) to detect cervical cancer in MRI images with a focus on deep imaging features critical for accurate classification. The MRI scans were conducted for various types of cancer, thereby providing adequate and diverse input to train deep learning models. They applied Learning Without Forgetting (LwF) to save knowledge from previous sets of data and improve the classification of new data. The top one was MobileNetV3 Small with 86% accuracy. Xception and Inception V3 architectures were also applied for further improvement, as the immense computational processing demands of MRI data were given top priority.

While previous studies have achieved promising results using deep learning in cervical cancer diagnosis, most of them rely on either single-modality data or fixed-weight fusion approaches. In contrast, our work introduces a novel hybrid framework that combines semantic and spatial feature extractors (InternImage and LVM) and applies dynamic fusion using SOA. This enables more adaptive learning across heterogeneous inputs. Furthermore, by using both CT and MRI modalities, our model overcomes limitations of modality-specific training seen in prior research. These innovations directly address the gaps identified in the literature and demonstrate a more clinically adaptable and technically robust approach.

## 5 Conclusions and feather work

When diagnosing cervical cancer patients, computed tomography (CT) and magnetic resonance imaging (MRI) are crucial. Imaging can identify the primary tumor, demonstrate local and distant disease progression, help define radiation fields, evaluate treatment efficacy, and facilitate tracking of disease relapse after treatment. Using deep learning techniques, the paper attempts to determine the efficiency of computed tomography (CT) and magnetic resonance imaging (MRI) as cancer imaging agents for cervical cancer. To classify between medical images (MRI and CT) as belonging to three classes: normal, benign, and malignant, the model used was the CNN-based LVM model, as well as InternImage based on InceptionV3. Output from both models was derived independently. For the improvement of classification accuracy, the Shark Optimization Algorithm (SOA) was used to optimize the performance of the two models. Cervical CT scans and MRI were categorized into three classes based on two new datasets, KAUH-CCTD and KAUH-CCMD, gathered from King Abdullah University Hospital (KAUH) in Jordan. The proposed model achieved the best performance in diagnosing CT images, with an accuracy of 98.49%, while it achieved an accuracy of 92.92% in diagnosing MRI images.

In the future, we hope to create a multimodal computer-aided design system for cervical cancers by combining pertinent CT and MRI information. In the upcoming version, additional photos will be included to improve clarity and balance and assist researchers in creating algorithms for identifying cervical tumors. A variety of datasets will be used to assess the suggested model’s efficacy. Furthermore, current and upcoming research suggests that computer vision models could help patients and physicians by increasing the effectiveness of diagnostic procedures, saving time, and speeding up the identification of benign and malignant cervical cancers. The current model’s architecture allows for clinical growth even though its primary function is to classify cervical pictures into three categories: benign, malignant, and normal. By adding more state-of-the-art deep learning models like ConvNeXt, Vision Transformer (ViT), Swin Transformer, and EfficientNetV2, we hope to broaden the comparative study. These architectures have demonstrated excellent performance in medical image processing and may shed more light on the possibilities of transformer-based and sophisticated CNN models for cervical cancer diagnosis. Furthermore, future research may use spatial feature extraction from LVM to infer tumor staging measures. Lastly, to improve clinical trust and support risk-based decision-making, uncertainty estimating techniques like Monte Carlo dropout can be used to generate confidence scores with every prediction.

## Data Availability

The datasets generated and analyzed during the current study are not publicly available due to institutional restrictions, but they can be made available from the corresponding author upon reasonable request at mohammadamin5111999@gmail.com. This study was conducted according to the guidelines and with the approval of the Institutional Review Board (IRB No. 21/171/2024) at King Abdullah University Hospital, Jordan University of Science and Technology, Jordan. Institutional Review Board approval has been granted.
